# Supplementary material for: Multi‐dataset identification of innovative feature genes and molecular mechanisms in keratoconus
Source: J Cell Mol Med. 2024 Sep 19;28(18):e70079. doi: 10.1111/jcmm.70079 (PMC11412914; doi:10.1111/jcmm.70079)
Supplement: Supplementary file 2 — Material S2. [file JCMM-28-e70079-s002.zip › 7.GSEA analysis/6.SNORA74B/KEGG_PATHWAYS_IN_CANCER.html]

Details for gene set KEGG\_PATHWAYS\_IN\_CANCER[GSEA]

|  || Dataset | SNORA74B.group.cls#h\_versus\_l |
| Phenotype | group.cls#h\_versus\_l |
| Upregulated in class | l |
| GeneSet | KEGG\_PATHWAYS\_IN\_CANCER |
| Enrichment Score (ES) | -0.3573211 |
| Normalized Enrichment Score (NES) | -1.5241202 |
| Nominal p-value | 0.019455252 |
| FDR q-value | 0.33139127 |
| FWER p-Value | 0.737 |
Table: GSEA Results Summary

  

Fig 1: Enrichment plot: KEGG\_PATHWAYS\_IN\_CANCER      
 Profile of the Running ES Score & Positions of GeneSet Members on the Rank Ordered List

  

| SYMBOL | TITLE | RANK IN GENE LIST | RANK METRIC SCORE | RUNNING ES | CORE ENRICHMENT || 1 | PIK3R5 | na | 170 | 0.800 | 0.0129 | No |
| 2 | FN1 | na | 587 | 0.575 | 0.0136 | No |
| 3 | RAC3 | na | 1795 | 0.390 | -0.0128 | No |
| 4 | FGF18 | na | 1820 | 0.388 | -0.0048 | No |
| 5 | RARB | na | 1895 | 0.380 | 0.0015 | No |
| 6 | LAMB4 | na | 2399 | 0.339 | -0.0055 | No |
| 7 | PRKCG | na | 2539 | 0.330 | -0.0022 | No |
| 8 | FGF8 | na | 2974 | 0.302 | -0.0081 | No |
| 9 | RET | na | 3274 | 0.288 | -0.0104 | No |
| 10 | FGF5 | na | 3327 | 0.285 | -0.0055 | No |
| 11 | FGF17 | na | 3644 | 0.272 | -0.0086 | No |
| 12 | PDGFRA | na | 4146 | 0.255 | -0.0175 | No |
| 13 | FLT3 | na | 4865 | 0.230 | -0.0332 | No |
| 14 | CBLB | na | 5386 | 0.212 | -0.0436 | No |
| 15 | FGF16 | na | 5815 | 0.198 | -0.0516 | No |
| 16 | SOS1 | na | 5971 | 0.193 | -0.0518 | No |
| 17 | IGF1R | na | 6399 | 0.180 | -0.0602 | No |
| 18 | DAPK1 | na | 6884 | 0.166 | -0.0706 | No |
| 19 | CCNE2 | na | 7159 | 0.158 | -0.0751 | No |
| 20 | COL4A1 | na | 7227 | 0.156 | -0.0735 | No |
| 21 | PTCH2 | na | 7369 | 0.152 | -0.0742 | No |
| 22 | KLK3 | na | 7466 | 0.149 | -0.0737 | No |
| 23 | FGF3 | na | 7481 | 0.149 | -0.0708 | No |
| 24 | TRAF3 | na | 7547 | 0.147 | -0.0694 | No |
| 25 | E2F2 | na | 7913 | 0.138 | -0.0769 | No |
| 26 | RALGDS | na | 8007 | 0.136 | -0.0766 | No |
| 27 | BRAF | na | 8089 | 0.134 | -0.0760 | No |
| 28 | PIK3R1 | na | 8116 | 0.133 | -0.0738 | No |
| 29 | PLCG2 | na | 8239 | 0.131 | -0.0744 | No |
| 30 | WNT16 | na | 8300 | 0.129 | -0.0733 | No |
| 31 | IGF1 | na | 8530 | 0.124 | -0.0771 | No |
| 32 | CCNA1 | na | 8695 | 0.120 | -0.0792 | No |
| 33 | SPI1 | na | 8726 | 0.120 | -0.0774 | No |
| 34 | IKBKB | na | 8763 | 0.119 | -0.0758 | No |
| 35 | MTOR | na | 8938 | 0.115 | -0.0783 | No |
| 36 | WNT10B | na | 8944 | 0.115 | -0.0759 | No |
| 37 | CCNE1 | na | 8992 | 0.114 | -0.0747 | No |
| 38 | CASP8 | na | 9153 | 0.110 | -0.0769 | No |
| 39 | LAMA5 | na | 9197 | 0.109 | -0.0758 | No |
| 40 | AKT2 | na | 9380 | 0.105 | -0.0787 | No |
| 41 | TGFBR2 | na | 9513 | 0.102 | -0.0802 | No |
| 42 | PTCH1 | na | 9661 | 0.099 | -0.0823 | No |
| 43 | PLCG1 | na | 9672 | 0.099 | -0.0804 | No |
| 44 | CEBPA | na | 9683 | 0.099 | -0.0785 | No |
| 45 | FASLG | na | 9725 | 0.098 | -0.0775 | No |
| 46 | KRAS | na | 9766 | 0.097 | -0.0765 | No |
| 47 | CASP3 | na | 9789 | 0.097 | -0.0749 | No |
| 48 | PDGFA | na | 9861 | 0.095 | -0.0749 | No |
| 49 | CDKN2B | na | 10073 | 0.091 | -0.0790 | No |
| 50 | MECOM | na | 10361 | 0.086 | -0.0854 | No |
| 51 | MAPK9 | na | 10427 | 0.085 | -0.0854 | No |
| 52 | WNT5A | na | 10441 | 0.084 | -0.0839 | No |
| 53 | AR | na | 10468 | 0.084 | -0.0828 | No |
| 54 | NTRK1 | na | 10524 | 0.083 | -0.0825 | No |
| 55 | CREBBP | na | 10525 | 0.083 | -0.0807 | No |
| 56 | LAMC3 | na | 10898 | 0.077 | -0.0898 | No |
| 57 | FGF20 | na | 10959 | 0.076 | -0.0899 | No |
| 58 | FGFR3 | na | 11176 | 0.072 | -0.0945 | No |
| 59 | CBL | na | 11198 | 0.072 | -0.0935 | No |
| 60 | MDM2 | na | 11211 | 0.071 | -0.0923 | No |
| 61 | PIAS1 | na | 11214 | 0.071 | -0.0908 | No |
| 62 | BID | na | 11264 | 0.070 | -0.0906 | No |
| 63 | CTBP1 | na | 11288 | 0.070 | -0.0897 | No |
| 64 | MSH3 | na | 11395 | 0.069 | -0.0913 | No |
| 65 | VHL | na | 11509 | 0.067 | -0.0931 | No |
| 66 | APPL1 | na | 11768 | 0.063 | -0.0992 | No |
| 67 | TGFB2 | na | 11803 | 0.063 | -0.0988 | No |
| 68 | RASSF5 | na | 12018 | 0.060 | -0.1036 | No |
| 69 | FGF9 | na | 12036 | 0.059 | -0.1028 | No |
| 70 | WNT8A | na | 12290 | 0.056 | -0.1089 | No |
| 71 | RXRA | na | 12303 | 0.056 | -0.1080 | No |
| 72 | ARNT | na | 12355 | 0.055 | -0.1083 | No |
| 73 | XIAP | na | 12442 | 0.054 | -0.1096 | No |
| 74 | WNT3 | na | 12537 | 0.053 | -0.1111 | No |
| 75 | TCF7L2 | na | 12663 | 0.051 | -0.1136 | No |
| 76 | PTEN | na | 12756 | 0.050 | -0.1152 | No |
| 77 | ERBB2 | na | 12767 | 0.050 | -0.1144 | No |
| 78 | HSP90AA1 | na | 12770 | 0.050 | -0.1133 | No |
| 79 | PIK3R2 | na | 12946 | 0.047 | -0.1173 | No |
| 80 | PIK3CA | na | 13079 | 0.046 | -0.1201 | No |
| 81 | FADD | na | 13177 | 0.045 | -0.1220 | No |
| 82 | SMAD4 | na | 13214 | 0.045 | -0.1220 | No |
| 83 | SMAD2 | na | 13222 | 0.045 | -0.1212 | No |
| 84 | CTBP2 | na | 13433 | 0.042 | -0.1264 | No |
| 85 | RB1 | na | 13606 | 0.040 | -0.1305 | No |
| 86 | SOS2 | na | 13685 | 0.039 | -0.1319 | No |
| 87 | FGF2 | na | 13739 | 0.039 | -0.1325 | No |
| 88 | RALBP1 | na | 13740 | 0.039 | -0.1317 | No |
| 89 | FGF11 | na | 13944 | 0.036 | -0.1368 | No |
| 90 | CBLC | na | 13962 | 0.036 | -0.1364 | No |
| 91 | RBX1 | na | 14044 | 0.035 | -0.1380 | No |
| 92 | WNT2 | na | 14134 | 0.034 | -0.1398 | No |
| 93 | TRAF4 | na | 14179 | 0.034 | -0.1404 | No |
| 94 | SUFU | na | 14239 | 0.033 | -0.1413 | No |
| 95 | FZD1 | na | 14343 | 0.032 | -0.1436 | No |
| 96 | STAT1 | na | 14481 | 0.030 | -0.1469 | No |
| 97 | PDGFRB | na | 14565 | 0.029 | -0.1487 | No |
| 98 | FGF22 | na | 14606 | 0.029 | -0.1492 | No |
| 99 | HHIP | na | 14705 | 0.028 | -0.1514 | No |
| 100 | MAP2K2 | na | 14904 | 0.025 | -0.1566 | No |
| 101 | MAPK10 | na | 14996 | 0.024 | -0.1587 | No |
| 102 | FGF13 | na | 15000 | 0.024 | -0.1583 | No |
| 103 | CDH1 | na | 15137 | 0.023 | -0.1617 | No |
| 104 | TRAF2 | na | 15165 | 0.023 | -0.1620 | No |
| 105 | STAT5B | na | 15213 | 0.022 | -0.1628 | No |
| 106 | CCND1 | na | 15223 | 0.022 | -0.1626 | No |
| 107 | MAX | na | 15362 | 0.021 | -0.1661 | No |
| 108 | FZD5 | na | 15466 | 0.020 | -0.1687 | No |
| 109 | NCOA4 | na | 15745 | 0.017 | -0.1764 | No |
| 110 | PIK3CB | na | 15769 | 0.017 | -0.1767 | No |
| 111 | CTNNA1 | na | 16086 | 0.014 | -0.1856 | No |
| 112 | BCR | na | 16207 | 0.013 | -0.1888 | No |
| 113 | SMAD3 | na | 16318 | 0.011 | -0.1917 | No |
| 114 | FZD6 | na | 16365 | 0.011 | -0.1928 | No |
| 115 | DAPK3 | na | 16384 | 0.011 | -0.1931 | No |
| 116 | CHUK | na | 16402 | 0.010 | -0.1934 | No |
| 117 | MAPK3 | na | 16753 | 0.007 | -0.2034 | No |
| 118 | HDAC1 | na | 16993 | 0.005 | -0.2102 | No |
| 119 | BIRC2 | na | 16995 | 0.005 | -0.2101 | No |
| 120 | E2F3 | na | 17092 | 0.004 | -0.2128 | No |
| 121 | FGF10 | na | 17212 | 0.003 | -0.2162 | No |
| 122 | FGF19 | na | 17258 | 0.002 | -0.2175 | No |
| 123 | EGF | na | 17351 | 0.001 | -0.2202 | No |
| 124 | CCDC6 | na | 17442 | 0.000 | -0.2228 | No |
| 125 | DAPK2 | na | 17464 | -0.000 | -0.2234 | No |
| 126 | PPARD | na | 17698 | -0.003 | -0.2301 | No |
| 127 | GSTP1 | na | 17706 | -0.003 | -0.2302 | No |
| 128 | CTNNB1 | na | 17783 | -0.003 | -0.2324 | No |
| 129 | RASSF1 | na | 17802 | -0.004 | -0.2328 | No |
| 130 | GSK3B | na | 17871 | -0.004 | -0.2347 | No |
| 131 | FH | na | 17939 | -0.005 | -0.2365 | No |
| 132 | SKP2 | na | 17943 | -0.005 | -0.2365 | No |
| 133 | JUP | na | 17968 | -0.006 | -0.2371 | No |
| 134 | RALB | na | 17970 | -0.006 | -0.2370 | No |
| 135 | FZD9 | na | 18101 | -0.007 | -0.2406 | No |
| 136 | RALA | na | 18156 | -0.008 | -0.2420 | No |
| 137 | WNT4 | na | 18195 | -0.008 | -0.2429 | No |
| 138 | RHOA | na | 18290 | -0.009 | -0.2454 | No |
| 139 | AKT1 | na | 18366 | -0.010 | -0.2474 | No |
| 140 | PLD1 | na | 18510 | -0.012 | -0.2513 | No |
| 141 | WNT3A | na | 18601 | -0.013 | -0.2536 | No |
| 142 | TPM3 | na | 18657 | -0.013 | -0.2549 | No |
| 143 | VEGFB | na | 18662 | -0.013 | -0.2547 | No |
| 144 | FZD3 | na | 18707 | -0.014 | -0.2557 | No |
| 145 | CDC42 | na | 18770 | -0.015 | -0.2572 | No |
| 146 | STK4 | na | 18825 | -0.015 | -0.2584 | No |
| 147 | CTNNA2 | na | 18831 | -0.015 | -0.2582 | No |
| 148 | PIK3CG | na | 18884 | -0.016 | -0.2594 | No |
| 149 | RAC2 | na | 18892 | -0.016 | -0.2592 | No |
| 150 | BAX | na | 18953 | -0.016 | -0.2606 | No |
| 151 | FOXO1 | na | 18961 | -0.017 | -0.2604 | No |
| 152 | PIAS4 | na | 19003 | -0.017 | -0.2612 | No |
| 153 | LAMA4 | na | 19146 | -0.019 | -0.2649 | No |
| 154 | BAD | na | 19265 | -0.021 | -0.2679 | No |
| 155 | DCC | na | 19371 | -0.022 | -0.2705 | No |
| 156 | HGF | na | 19424 | -0.022 | -0.2715 | No |
| 157 | FGF14 | na | 19449 | -0.022 | -0.2717 | No |
| 158 | TP53 | na | 19572 | -0.024 | -0.2747 | No |
| 159 | EGFR | na | 19596 | -0.024 | -0.2748 | No |
| 160 | HRAS | na | 19699 | -0.025 | -0.2772 | No |
| 161 | HSP90AB1 | na | 19743 | -0.026 | -0.2779 | No |
| 162 | CDKN2A | na | 19773 | -0.026 | -0.2782 | No |
| 163 | BIRC5 | na | 19815 | -0.026 | -0.2788 | No |
| 164 | FOS | na | 19844 | -0.027 | -0.2790 | No |
| 165 | RAC1 | na | 19881 | -0.027 | -0.2794 | No |
| 166 | RARA | na | 20023 | -0.029 | -0.2829 | No |
| 167 | WNT9A | na | 20087 | -0.029 | -0.2841 | No |
| 168 | CDKN1B | na | 20194 | -0.030 | -0.2865 | No |
| 169 | LAMB2 | na | 20349 | -0.032 | -0.2902 | No |
| 170 | PIK3CD | na | 20354 | -0.032 | -0.2896 | No |
| 171 | MSH2 | na | 20390 | -0.033 | -0.2899 | No |
| 172 | GLI3 | na | 20392 | -0.033 | -0.2892 | No |
| 173 | MAPK8 | na | 20414 | -0.033 | -0.2891 | No |
| 174 | FGF12 | na | 20421 | -0.033 | -0.2885 | No |
| 175 | FGF7 | na | 20472 | -0.034 | -0.2892 | No |
| 176 | MAPK1 | na | 20606 | -0.035 | -0.2923 | No |
| 177 | IKBKG | na | 20820 | -0.038 | -0.2977 | No |
| 178 | AXIN1 | na | 20829 | -0.038 | -0.2971 | No |
| 179 | DVL3 | na | 20946 | -0.039 | -0.2996 | No |
| 180 | TGFA | na | 20989 | -0.040 | -0.2999 | No |
| 181 | RAF1 | na | 21074 | -0.041 | -0.3014 | No |
| 182 | PIAS3 | na | 21552 | -0.046 | -0.3142 | No |
| 183 | JAK1 | na | 21809 | -0.049 | -0.3206 | No |
| 184 | CASP9 | na | 21843 | -0.050 | -0.3204 | No |
| 185 | DVL1 | na | 21883 | -0.050 | -0.3204 | No |
| 186 | TFG | na | 22067 | -0.053 | -0.3246 | No |
| 187 | ITGA2 | na | 22079 | -0.053 | -0.3237 | No |
| 188 | HDAC2 | na | 22146 | -0.053 | -0.3245 | No |
| 189 | PRKCA | na | 22239 | -0.055 | -0.3259 | No |
| 190 | CYCS | na | 22291 | -0.055 | -0.3262 | No |
| 191 | TRAF6 | na | 22560 | -0.059 | -0.3326 | No |
| 192 | CSF3R | na | 22572 | -0.059 | -0.3316 | No |
| 193 | APC2 | na | 22646 | -0.060 | -0.3324 | No |
| 194 | PTK2 | na | 22693 | -0.061 | -0.3324 | No |
| 195 | COL4A2 | na | 22912 | -0.064 | -0.3373 | No |
| 196 | MSH6 | na | 22913 | -0.064 | -0.3359 | No |
| 197 | PML | na | 23015 | -0.066 | -0.3374 | No |
| 198 | PAX8 | na | 23291 | -0.070 | -0.3438 | No |
| 199 | PIAS2 | na | 23327 | -0.070 | -0.3432 | No |
| 200 | ITGA3 | na | 23349 | -0.071 | -0.3423 | No |
| 201 | KIT | na | 23499 | -0.073 | -0.3450 | No |
| 202 | FZD4 | na | 23557 | -0.074 | -0.3450 | No |
| 203 | ABL1 | na | 23665 | -0.076 | -0.3464 | No |
| 204 | ITGA2B | na | 23836 | -0.078 | -0.3496 | No |
| 205 | LAMB1 | na | 23959 | -0.080 | -0.3514 | No |
| 206 | LAMA2 | na | 24013 | -0.081 | -0.3511 | No |
| 207 | MLH1 | na | 24023 | -0.081 | -0.3496 | No |
| 208 | FLT3LG | na | 24043 | -0.081 | -0.3483 | No |
| 209 | EGLN2 | na | 24126 | -0.083 | -0.3488 | No |
| 210 | CDK4 | na | 24176 | -0.084 | -0.3484 | No |
| 211 | FGFR2 | na | 24244 | -0.085 | -0.3485 | No |
| 212 | EGLN1 | na | 24335 | -0.086 | -0.3492 | No |
| 213 | CKS1B | na | 24340 | -0.086 | -0.3474 | No |
| 214 | HSP90B1 | na | 24525 | -0.090 | -0.3507 | No |
| 215 | RXRB | na | 24595 | -0.091 | -0.3507 | No |
| 216 | FGF6 | na | 24652 | -0.092 | -0.3502 | No |
| 217 | BIRC3 | na | 24774 | -0.094 | -0.3517 | No |
| 218 | EPAS1 | na | 24835 | -0.095 | -0.3513 | No |
| 219 | STK36 | na | 24838 | -0.096 | -0.3492 | No |
| 220 | GRB2 | na | 24997 | -0.098 | -0.3516 | No |
| 221 | VEGFA | na | 25195 | -0.102 | -0.3550 | Yes |
| 222 | FGF1 | na | 25243 | -0.103 | -0.3541 | Yes |
| 223 | HIF1A | na | 25313 | -0.104 | -0.3538 | Yes |
| 224 | WNT2B | na | 25314 | -0.104 | -0.3515 | Yes |
| 225 | JUN | na | 25317 | -0.104 | -0.3492 | Yes |
| 226 | EP300 | na | 25385 | -0.105 | -0.3488 | Yes |
| 227 | CTNNA3 | na | 25470 | -0.107 | -0.3489 | Yes |
| 228 | MMP2 | na | 25525 | -0.108 | -0.3481 | Yes |
| 229 | MYC | na | 25534 | -0.108 | -0.3459 | Yes |
| 230 | FZD8 | na | 25688 | -0.111 | -0.3479 | Yes |
| 231 | KITLG | na | 25692 | -0.111 | -0.3455 | Yes |
| 232 | TRAF5 | na | 25981 | -0.117 | -0.3513 | Yes |
| 233 | LAMC2 | na | 26097 | -0.119 | -0.3520 | Yes |
| 234 | BRCA2 | na | 26233 | -0.122 | -0.3532 | Yes |
| 235 | SLC2A1 | na | 26243 | -0.122 | -0.3507 | Yes |
| 236 | STAT3 | na | 26362 | -0.124 | -0.3514 | Yes |
| 237 | PTGS2 | na | 26424 | -0.126 | -0.3503 | Yes |
| 238 | NFKB1 | na | 26534 | -0.128 | -0.3506 | Yes |
| 239 | DVL2 | na | 26618 | -0.130 | -0.3502 | Yes |
| 240 | CUL2 | na | 26634 | -0.131 | -0.3477 | Yes |
| 241 | ITGAV | na | 26724 | -0.133 | -0.3473 | Yes |
| 242 | CRK | na | 26740 | -0.134 | -0.3447 | Yes |
| 243 | WNT10A | na | 26979 | -0.139 | -0.3486 | Yes |
| 244 | CDK2 | na | 27098 | -0.141 | -0.3488 | Yes |
| 245 | FGF21 | na | 27099 | -0.142 | -0.3457 | Yes |
| 246 | LAMC1 | na | 27122 | -0.142 | -0.3432 | Yes |
| 247 | TGFBR1 | na | 27137 | -0.142 | -0.3404 | Yes |
| 248 | TCF7L1 | na | 27185 | -0.144 | -0.3385 | Yes |
| 249 | E2F1 | na | 27392 | -0.149 | -0.3412 | Yes |
| 250 | ITGB1 | na | 27543 | -0.152 | -0.3422 | Yes |
| 251 | CDK6 | na | 27632 | -0.155 | -0.3413 | Yes |
| 252 | SMO | na | 27678 | -0.156 | -0.3391 | Yes |
| 253 | GLI2 | na | 27694 | -0.157 | -0.3361 | Yes |
| 254 | FZD10 | na | 27856 | -0.161 | -0.3371 | Yes |
| 255 | TPR | na | 27863 | -0.161 | -0.3337 | Yes |
| 256 | LAMA3 | na | 27891 | -0.162 | -0.3309 | Yes |
| 257 | ARNT2 | na | 27894 | -0.162 | -0.3273 | Yes |
| 258 | ARAF | na | 27916 | -0.162 | -0.3243 | Yes |
| 259 | FAS | na | 27948 | -0.163 | -0.3216 | Yes |
| 260 | ITGA6 | na | 27950 | -0.163 | -0.3180 | Yes |
| 261 | NRAS | na | 27969 | -0.164 | -0.3148 | Yes |
| 262 | CSF1R | na | 28378 | -0.176 | -0.3228 | Yes |
| 263 | NFKB2 | na | 28383 | -0.176 | -0.3190 | Yes |
| 264 | NFKBIA | na | 28570 | -0.182 | -0.3203 | Yes |
| 265 | STAT5A | na | 28673 | -0.185 | -0.3192 | Yes |
| 266 | MET | na | 28809 | -0.189 | -0.3189 | Yes |
| 267 | TCF7 | na | 28810 | -0.189 | -0.3147 | Yes |
| 268 | BCL2L1 | na | 28814 | -0.189 | -0.3105 | Yes |
| 269 | WNT7B | na | 28850 | -0.190 | -0.3073 | Yes |
| 270 | LAMB3 | na | 29125 | -0.198 | -0.3108 | Yes |
| 271 | CRKL | na | 29271 | -0.203 | -0.3105 | Yes |
| 272 | PIK3R3 | na | 29453 | -0.208 | -0.3112 | Yes |
| 273 | RELA | na | 29651 | -0.214 | -0.3121 | Yes |
| 274 | WNT8B | na | 29836 | -0.219 | -0.3126 | Yes |
| 275 | APC | na | 30020 | -0.226 | -0.3128 | Yes |
| 276 | COL4A4 | na | 30027 | -0.227 | -0.3079 | Yes |
| 277 | PDGFB | na | 30041 | -0.227 | -0.3033 | Yes |
| 278 | TGFB1 | na | 30144 | -0.231 | -0.3011 | Yes |
| 279 | FGF23 | na | 30156 | -0.231 | -0.2962 | Yes |
| 280 | PGF | na | 30172 | -0.232 | -0.2915 | Yes |
| 281 | WNT9B | na | 30647 | -0.250 | -0.2997 | Yes |
| 282 | RUNX1 | na | 31103 | -0.273 | -0.3068 | Yes |
| 283 | MAP2K1 | na | 31129 | -0.275 | -0.3014 | Yes |
| 284 | CDKN1A | na | 31225 | -0.280 | -0.2979 | Yes |
| 285 | CXCL8 | na | 31337 | -0.285 | -0.2948 | Yes |
| 286 | TGFB3 | na | 31437 | -0.291 | -0.2912 | Yes |
| 287 | RAD51 | na | 31546 | -0.298 | -0.2877 | Yes |
| 288 | PPARG | na | 31596 | -0.300 | -0.2824 | Yes |
| 289 | EGLN3 | na | 31600 | -0.301 | -0.2758 | Yes |
| 290 | WNT7A | na | 31601 | -0.301 | -0.2691 | Yes |
| 291 | RUNX1T1 | na | 32078 | -0.327 | -0.2757 | Yes |
| 292 | IL6 | na | 32098 | -0.328 | -0.2689 | Yes |
| 293 | WNT1 | na | 32208 | -0.335 | -0.2646 | Yes |
| 294 | WNT6 | na | 32236 | -0.336 | -0.2579 | Yes |
| 295 | FZD2 | na | 32364 | -0.345 | -0.2539 | Yes |
| 296 | CSF2RA | na | 32416 | -0.349 | -0.2476 | Yes |
| 297 | WNT5B | na | 32480 | -0.353 | -0.2416 | Yes |
| 298 | TRAF1 | na | 32584 | -0.360 | -0.2365 | Yes |
| 299 | GLI1 | na | 32735 | -0.371 | -0.2326 | Yes |
| 300 | ZBTB16 | na | 32824 | -0.378 | -0.2267 | Yes |
| 301 | ETS1 | na | 33147 | -0.409 | -0.2270 | Yes |
| 302 | COL4A6 | na | 33172 | -0.411 | -0.2185 | Yes |
| 303 | BMP4 | na | 33250 | -0.419 | -0.2114 | Yes |
| 304 | FZD7 | na | 33265 | -0.421 | -0.2025 | Yes |
| 305 | BMP2 | na | 33393 | -0.437 | -0.1964 | Yes |
| 306 | MMP9 | na | 33562 | -0.458 | -0.1911 | Yes |
| 307 | RXRG | na | 33623 | -0.467 | -0.1824 | Yes |
| 308 | LAMA1 | na | 33685 | -0.475 | -0.1736 | Yes |
| 309 | BCL2 | na | 33746 | -0.485 | -0.1645 | Yes |
| 310 | PRKCB | na | 33882 | -0.508 | -0.1571 | Yes |
| 311 | SHH | na | 33951 | -0.524 | -0.1474 | Yes |
| 312 | AXIN2 | na | 34084 | -0.552 | -0.1389 | Yes |
| 313 | MMP1 | na | 34117 | -0.561 | -0.1274 | Yes |
| 314 | NOS2 | na | 34182 | -0.577 | -0.1164 | Yes |
| 315 | VEGFC | na | 34267 | -0.599 | -0.1054 | Yes |
| 316 | FGF4 | na | 34349 | -0.632 | -0.0937 | Yes |
| 317 | LEF1 | na | 34351 | -0.633 | -0.0796 | Yes |
| 318 | NKX3-1 | na | 34408 | -0.652 | -0.0667 | Yes |
| 319 | WNT11 | na | 34430 | -0.665 | -0.0525 | Yes |
| 320 | AKT3 | na | 34657 | -0.880 | -0.0394 | Yes |
| 321 | MITF | na | 34665 | -0.895 | -0.0197 | Yes |
| 322 | FGFR1 | na | 34687 | -0.963 | 0.0012 | Yes |
Table: GSEA details [plain text format]

  

Fig 2: KEGG\_PATHWAYS\_IN\_CANCER      
 Blue-Pink O' Gram in the Space of the Analyzed GeneSet

  

Fig 3: KEGG\_PATHWAYS\_IN\_CANCER: Random ES distribution      
 Gene set null distribution of ES for **KEGG\_PATHWAYS\_IN\_CANCER**
